# Enhanced Light Extraction of Flip-Chip Mini-LEDs with Prism-Structured Sidewall

**DOI:** 10.3390/nano9030319

**Published:** 2019-02-28

**Authors:** Bin Tang, Jia Miao, Yingce Liu, Hui Wan, Ning Li, Shengjun Zhou, Chengqun Gui

**Affiliations:** 1 Key Laboratory of Hydraulic Machinery Transients (Wuhan University), Ministry of Education, Wuhan 430072, China; bintang@whu.edu.cn (B.T.); miaojia789@whu.edu.cn (J.M.); liningmick@whu.edu.cn (N.L.); 2 Xiamen Changelight Co. Ltd., Xiamen 361000, China; tigerlyc@163.com; 3 The Institute of Technological Sciences, Wuhan University, Wuhan 430072, China; wanhui_hb@whu.edu.cn (H.W.); cheng.gui.2000@gmail.com (C.G.); 4 Center for Photonics and Semiconductors, School of Power and Mechanical Engineering, Wuhan University, Wuhan 430072, China; 5 State Key Laboratory of Applied Optics, Changchun Institute of Optics, Fine Mechanics and Physics, Chinese Academy of Sciences, Changchun 130033, China

**Keywords:** flip-chip mini-LED, prism-structured sidewall, waveguide photons, light extraction

## Abstract

Current solutions for improving the light extraction efficiency of flip-chip light-emitting diodes (LEDs) mainly focus on relieving the total internal reflection at sapphire/air interface, but such methods hardly affect the epilayer mode photons. We demonstrated that the prism-structured sidewall based on tetramethylammonium hydroxide (TMAH) etching is a cost-effective solution for promoting light extraction efficiency of flip-chip mini-LEDs. The anisotropic TMAH etching created hierarchical prism structure on sidewall of mini-LEDs for coupling out photons into air without deteriorating the electrical property. Prism-structured sidewall effectively improved light output power of mini-LEDs by 10.3%, owing to the scattering out of waveguided light trapped in the gallium nitride (GaN) epilayer.

## 1. Introduction

The developments in GaN-based light-emitting diodes (LEDs) have promoted the liquid crystal display (LCD) as a highly competitive display technology in the past few decades [1,2]. Recently, the application of mini-LEDs with size below 200 microns gains new advantages for LCDs in market competition owing to their prominent merits as backlight unit, such as long life span, low energy consumption and high resolution [3,4]. However, to meet the high dynamic range (HDR) requirements of next generation displays, the luminance of the LCD bright state should be over 1000 nits, which requests the mini-LED backlight unit to be much more energy efficient [5].

Tremendous efforts have been done to improve the efficiency of GaN-based LEDs, which can be principally divided into two categories: improving the crystal quality of epilayer [6,7,8,9,10] and boosting the light extraction efficiency (LEE) [11,12,13,14,15]. Since mini-LEDs are obtained from the identical epilayer as broad-area LEDs, the fruitful methods for high crystal quality epilayer are universal in fabrication of the two kinds of LEDs. Since the ratio of top emitting area to sidewall emitting area is greatly different for mini-LEDs and broad-area LEDs, the methods applicable in broad-area LEDs for improved LEE need to be reassessed in mini-LEDs.

Since the top surface area of mini-LED is much reduced, flip-chip structure is the preferable choice for mini-LEDs to ensure enough top emitting area as well as p-contact area. Owing to the index mismatch of GaN epilayer, sapphire substrate and air, the majority of photons are trapped in the high-index epilayer and sapphire substrate by total internal reflection (TIR) and guided laterally as waveguide modes, which finally dissipate in the lossy epilayer. Several methods have been proposed to extract the waveguide photons of flip-chip LEDs, such as texturing the sapphire subtract surface [16,17,18], shaping the sapphire substrate [19,20], plasmonic structure [21], and depositing nanoparticles or scattering layers [22,23]. Such approaches are effective in extraction of the sapphire substrate mode photons, while additional protection procedures are generally needed owing to the more inert property of sapphire substrate relative to the epilayer. More importantly, these approaches have no effect on epilayer mode photons. Simulation and experimental results have shown that reducing the pattern size of patterned sapphire substrate (PSS) is effective in improving the LEE by scattering out epilayer mode photons [24,25]. However, small pattern size is disadvantage for crystalline quality of epilayer [26] and the minimal spacing of PSS achieved in practice is far from the best outcoupling spacing for light extraction [27]. Thus, further works are still needed to extract the epilayer mode photons of flip-chip LEDs.

In this study, we demonstrated a simple and reliable method to extract the epilayer mode photons. Prism-structured sidewall generated by tetramethylammonium hydroxide (TMAH)-based crystallographic etching was introduced to scatter out epilayer mode photons. The size of prism structure on the sidewall of mini-LED could be manipulated from nanoscale to a few microns by adjusting TMAH etching time to achieve the best outcoupling efficiency. The anisotropic TMAH etching is damage-free and practical in mass-production, which makes the prism-structured sidewall based on TMAH etching a promising solution for highly efficient mini-LEDs.

## 2. Materials and Methods

**Figure 1:**
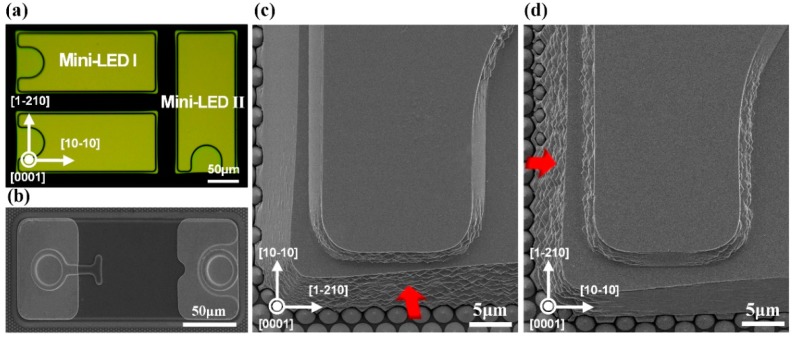
(**a**) Optical microscope image of the epilayer after ICP procedure, showing the orthogonal arrangements of mini-LED I and mini-LED II. (**b**) SEM image of the fabricated flip-chip mini-LED with a bird’s eye view, showing the dimension of the flip-chip mini-LED. (**c**) SEM image of the mini-LED I after TMAH etching treatment, the red arrow in the image points to the prism-structured sidewall. (**d**) SEM image of the mini-LED II after TMAH etching treatment, the red arrow in the image points to the prism-structured sidewall.

The GaN-based LEDs were grown on c-plane PSS using metal–organic chemical vapor deposition (MOCVD) method. The LED epitaxial structure consisted of a 25-nm-thick low temperature GaN nucleation layer, a 3.0-μm-thick undoped GaN buffer layer, a 2.5-μm-thick Si-doped n-GaN layer, a 12-pair of InGaN (3 nm)/GaN (12 nm) multiple quantum well (MQW), a 40-nm-thick p-AlGaN electron blocking layer, and a 112-nm-thick Mg-doped p-GaN layer. The LED wafer was subsequently annealed at 750 °C at N_2_ atmosphere to activate Mg acceptor in the p-GaN. Then, the photolithography and inductively coupled plasma etching (ICP) process based on BCl_3_/Cl_2_ mixture gas were performed to form the mesa structure and deep isolation trench. Afterwards, the TMAH-based crystallographic etching procedure was applied. The LED wafers were dipped into the 15 wt% TMAH solution at 85 °C during the TMAH etching process. After rinsing with deionized water and drying under N_2_ flow, a 60-nm-thick indium tin oxide (ITO) transparent conductive layer was evaporated on the p-GaN layer. Cr/Al/Ti/Pt/Au metallization schemed as ohmic contact layer was deposited on the ITO and n-GaN layer. Sixteen pairs of quarter-wavelength-thick TiO_2_/SiO_2_ stacks, as distributed Bragg reflectors, were sputtered by ion beam deposition followed by the opening of via through DBR using ICP etching based on CHF_3_/Ar/O_2_ mixture gas. Cr/Ti/Pt/Au metallization was evaporated as contact pads subsequently. Finally, the LED wafer was thinned down to about 150 μm and diced into chips with dimensions of 101 μm × 200 μm. The peak emission wavelength of the fabricated mini-LEDs was 456 nm. The light output power–current–voltage (*L-I-V*) characteristics of mini-LEDs were measured using a semiconductor parameter analyzer (Keysight B2901A, Santa Rosa, CA, USA) with an integrating sphere. In this work, two types of mini-LED chips with different sidewall orientations on the same LED wafer (as shown in Figure 1) were investigated by the scanning electron microscope (SEM) owing to the anisotropic etching behavior of TMAH-based crystallographic etching. The two types of mini-LEDs were named as mini-LED I and mini-LED II according to the sidewall orientation. The larger sidewalls of mini-LED I were set to be orientated along [10-10] direction, while the larger sidewalls of mini-LED II were set to be orientated along [1-210] direction.

## 3. Results and Discussion

**Figure 2:**
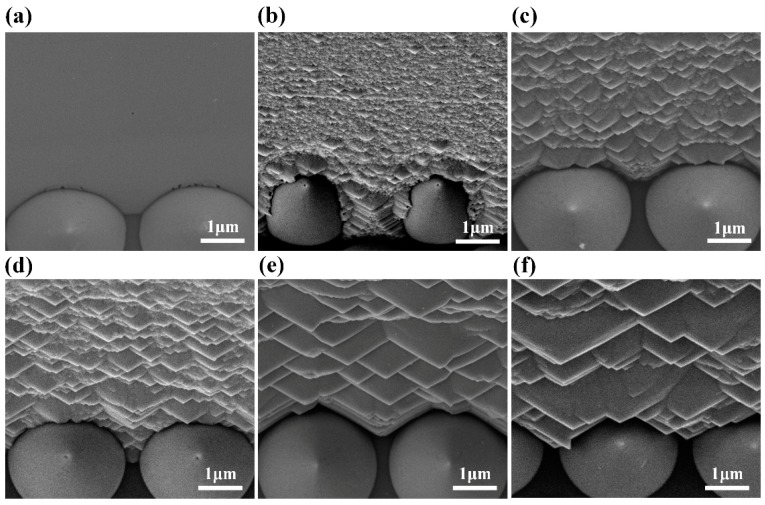
(**a**–**f**) SEM images of the chip sidewall along [1-210] direction with various TMAH etching time: (**a**) with 0 min TMAH etching treatment; (**b**) with 2.5 min TMAH etching treatment; (**c**) with 5 min TMAH etching treatment; (**d**) with 7.5 min TMAH etching treatment; (**e**) with 10 min TMAH etching treatment; and (**f**) with 20 min TMAH etching treatment.

We took advantage of the anisotropic etching behavior of TMAH-based crystallographic etching to obtain the textured sidewall structure. No additional protection procedure was incorporated owing to the damage-free and anisotropic etching properties of TMAH-based crystallographic etching, making it a convenient and cost effective solution to texture the sidewall of GaN epilayer. The selective etching ability of TMAH solution arises from the difference in density of N dangling bonds on different GaN lattice planes [28]. Surfaces with high density of N dangling bonds possess large repulsion to the hydroxide ions, which stops the crystallographic etching at such surface [29]. The density of N dangling bonds on different GaN lattice planes can be ranked as follows: (0001) plane > (1-210) plane > (10-10) plane > (000-1) plane [30]. Thus, the (0001) plane and (1-210) plane have larger repulsion force to the hydroxide ions, and the TMAH etching did not proceed on the top surface and the sidewall along [10-10] direction under our experiment condition. As shown in Figure 1c,d, no prism structure appeared on the top surface and the sidewall along [10-10] direction while the hierarchical prism structure appeared throughout the sidewall along [1-210] direction. Moreover, the textured sidewall surface area was different for the two types of mini-LEDs investigated owing to their orthogonal arrangements, which resulted in discrepant light output power as demonstrated by the *L-I-V* characteristics. The surface morphology of the sidewall along [1-210] direction with different TMAH etching time was characterized by SEM, as shown in Figure 2. Owing to the anisotropic etching property, the smooth surface was left with hierarchical prism structure after TMAH treatment. Trigonal prisms close to the PSS presented larger size than that in other regions, suggesting a larger TMAH etching rate near the interface of PSS and GaN. The larger etching rate may arise from that the TMAH etching started from the (000-1) plane at the interface. Within the etching time investigated, the TMAH etching proceeded at selected lattice planes and trigonal prism structures varied from nanoscale to a few microns as the TMAH etching time increased. The influence of prism size on light extraction is discussed below.

**Figure 3:**
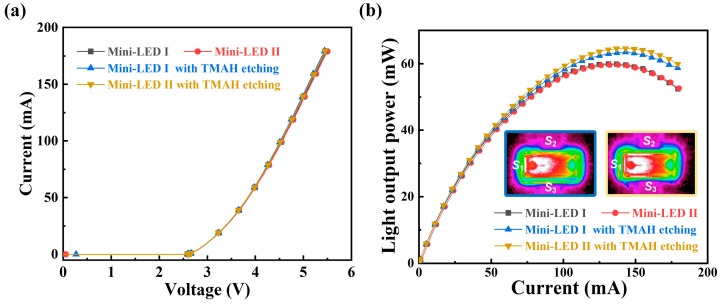
(**a**) *I-V* curves of the investigated two types of mini-LEDs with and without TMAH etching treatment. (**b**) *L-I* curves of the fabricated mini-LEDs with and without 7.5 min TMAH etching treatment. The insets show the photographs of TMAH treated LEDs under 10 mA injection current: mini-LED I with TMAH etching (**left**) and mini-LED II with TMAH etching (**right**).

To verify the TMAH etching is a damage-free process, the *I-V* characteristics of mini-LEDs with and without TMAH etching treatment were investigated, as shown in Figure 3a. A 7.5 min TMAH etching procedure only caused slight variation in forward voltages of mini-LEDs, suggesting no electrical degradation was brought in by the TMAH etching process. Previous reports on improving the sidewall surface area by ICP etching generally incorporate plasma damage, which leads to deteriorated electrical property [31,32]. The TMAH etching demonstrated here provided an alternative solution without reducing the top emitting area and deteriorating the electrical property.

**Figure 4:**
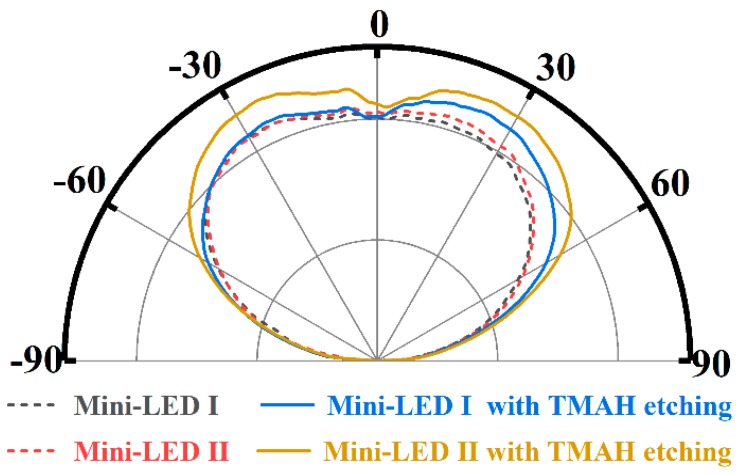
Far-field radiation patterns of the flip-chip mini-LEDs without TMAH etching treatment and with 7.5 min TMAH etching treatment.

Figure 3b shows the *L-I* curves of the investigated mini-LEDs, and the insets are photographs of TMAH treated mini-LEDs under 10 mA injection current. The *L-I* characteristics for mini-LED I and mini-LED II almost overlapped since they were fabricated from the same wafer. With injection current of 120 mA, the light output powers of the TMAH treated mini-LED I and TMAH treated mini-LED II were 62.5 mW and 65.6 mW, which were improved by 4.5% and 10.3% as compared to the mini-LEDs without TMAH treatment. The inset photographs show obvious brightness difference at the sidewall regions for the two types of mini-LEDs with TMAH etching. Mini-LED I with TMAH etching showed brighter *S*_1_ sidewall while mini-LED II with TMAH etching showed brighter *S*_2_ and *S*_3_ sidewall, corresponding to their prism-structured sidewalls. According to the far-field radiation patterns shown in Figure 4, the light emission of TMAH treated mini-LEDs from the side direction was significantly improved as compared to the mini-LEDs without TMAH treatment, while only slight variation along the surface normal direction was observed for the investigated mini-LEDs. These results suggest that the light output power enhancement of mini-LEDs with TMAH treatment can be mainly attributed to increased light extraction from the prism-structured sidewall surfaces.

**Figure 5:**
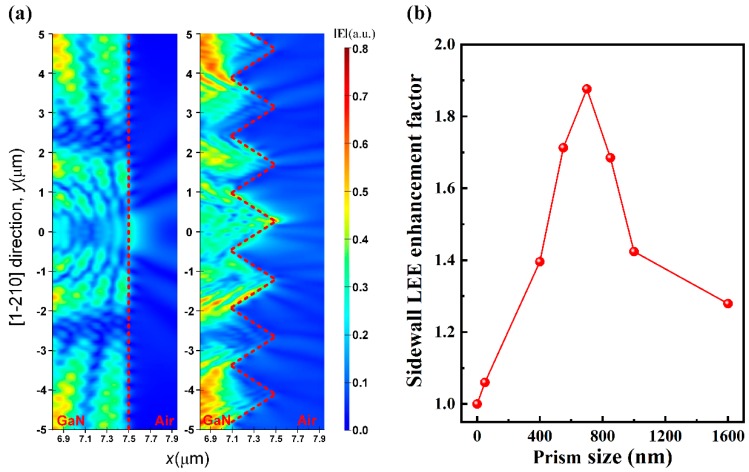
(**a**) Normalized electric field intensity distribution nearby the smooth (**left**) and prism-structured sidewalls (**right**) of LED chips from FDTD simulations. The color scale measures the electric field intensity E. (**b**) Simulated dependence of single sidewall light extraction efficiency on prism size parameters.

To reveal the fundamental principle of prism structure on light extraction, the finite-difference time-domain (FDTD) simulation was conducted. The simulation model was built based on the above-described device structure and scaled to the size of 15 μm × 30 μm considering the computational capacity. Perfectly matched layers (PML) was adopted as boundaries to avoid unnecessary reflected light. The grid size in the simulation domain was 10 nm for accuracy with the limitation of computer memory. Transverse electric (TE) and transverse magnetic (TM) polarized point sources with a ratio of 1.8:1 were positioned in the center region of MQW [33] and the emission wavelength was set to be 456 nm. Figure 5a shows the simulated electric field intensity distribution nearby the smooth and prism-structured sidewalls of epilayer. The electric field emitting out from the smooth sidewall is mainly confined at the center region, while an intensified electric field emitted out from the prism-structured sidewall with a broader distribution in air. The broader and stronger electric field emitting out from the prism-structured sidewall suggested that the prism-structured sidewall acted more effectively in extracting light out than the smooth sidewall. The relationship between sidewall light extraction efficiency and prism size is presented in Figure 5b. The strong dependence of sidewall light extraction efficiency on the prism size indicating that the enhancement mainly arose from more light scattering out from the epilayer mode rather than only randomizing of light rays [34].

## 4. Conclusions

In summary, we demonstrated the prism-structured sidewall based on TMAH etching as an effective approach for scattering out waveguided light from the GaN epilayer. After TMAH etching procedure, hierarchical prism structure generated on the sidewall along [1-210] direction without bringing in damages on other surfaces owing to the anisotropic property of TMAH etching. Compared to the control mini-LEDs, the light output power of mini-LEDs with prism-structured sidewall improved by 4.5% or 10.3%, respectively, according to the different arrangements of LED chips on the wafer. We suggest the cost-effective sidewall texturing approach proposed in this work is a promising way to realize high-efficiency flip-chip mini-LEDs.
